# A Facile Approach to High Precision Detection of Cell-to-Cell Variation for Li-ion Batteries

**DOI:** 10.1038/s41598-020-64174-2

**Published:** 2020-04-28

**Authors:** Leqiong Xie, Dongsheng Ren, Li Wang, Zonghai Chen, Guangyu Tian, Khalil Amine, Xiangming He

**Affiliations:** 1 Institute of Nuclear and New Energy Technology, Tsinghua University, Beijing, 100084 China; 2 Chemical Sciences and Engineering Division, Argonne National Laboratory, Argonne, IL 60439 USA; 3 State Key Laboratory of Automotive Safety and Energy, Tsinghua University, Beijing, 100084 China

**Keywords:** Batteries, Batteries, Characterization and analytical techniques, Characterization and analytical techniques

## Abstract

Over the past decade, it has been repeatedly demonstrated that homogeneity in electrochemical performance of lithium-ion cells plays a major role in determining the life and safety of lithium-ion battery modules or packs. Generally, the homogeneity of a battery pack is evaluated by characterizing the cells individually in terms of capacity, mass, impedance. Particularly, high quality electrochemical data heavily relies on the availability of high precision current source to minimize the discrepancy induced by the channel-to-channel variation. Here, a facile and precise measurement method is reported for screening cell-to-cell variations, in which voltage is the only indicator parameter independent of high precision current source. In detail, by connecting the cells in series (CiS), the measurement error of electrochemical data caused by stability and discrepancy of current sources among different charge/discharge equipment can be effectively avoided. The findings of this work showed that the cell-to-cell variations can be simply and sensitively detected with CiS configuration. For example, the relative standard deviation, which is the evaluation criterion of battery homogeneity, was 2.14% based on CiS while it was 0.43% based on individual measurements. The simple and precise CiS measurement is promising for evaluation of cell quality or module integration quality. In addition, this work can also provide a solid foundation for the development of detection algorithms for battery management systems to rapidly monitor battery homogeneity.

## Introduction

Lithium ion batteries (LIBs) have to be integrated into modules and packs for large-scale applications such as electric vehicles (EVs) and stationary energy storage systems ^1–7^. However, a reliable and long-lasting power system is determined mostly by the cell homogeneity rather than the performance of individual cells^8,9^. The cell-to-cell variations in terms of capacity and impedance will subject individual cells to different levels of state of charge (SOC), current, temperature and aging, which in turn would accelerate the degradation of electrochemical performance or even lead to safety performance of the power system^2,10–13^. For instance, a weak cell with a smaller capacity than the average in a series-connected module will be repeatedly overcharged (over-discharged) during charging (discharging) process if the charge/discharge cutoff condition is determined according to the total voltage^14^. The weaker cell will then have faster decay than others and will be susceptible to failure under harsh conditions. The available energy of the module will also be limited by the weakest cell^15^. Therefore, there is an urgent need to understand and minimize the cell-to-cell variations in a battery module.

There are different factors leading to variations among cells^11^, including manufacturing factors (manufacturing tolerances, quality control and process design), and environmental factors such as temperature gradient within the battery pack^16^. Rumpf *et al*.^12^ has evaluated the cell-to-cell variation due to manufacturing tolerances based on tests on 1100 commercial lithium-ion cells, and found relative variations of 0.28% and 0.72% for the cell capacity and impedance, respectively. Dubarry *et al*.^17^ has showed that cell-to-cell variations can drastically affect the performance and the reliability of both series and parallel battery packs. Baumhöfer *et al*.^18^ and Harris *et al*.^19^ showed that variation caused by production will result in a spread in battery degradation performance. Furthermore, theoretical analysis and industrial practice have showed that inhomogeneity of electrochemical characteristics in the unmatched cells inevitably resulted in voltage/SOC variations which can cause permanent internal damage in battery packs and energy storage systems^13,20^.

The energy loss and SOC estimation error can be minimized by reducing the cell-to-cell variation^13,21^. In order to achieve a stable configuration of a battery module or pack, a screening process is needed to ensure high homogeneity of the cells therein ^11,22,23^. Several screening processes have been previously proposed^12,20,24–26^. Multiple parameters, including capacity, mass, electrochemical impedance, direct current resistance and voltage, were employed to index the cell-to-cell variation. Rumpf *et al*.^12^ conducted a statistical analysis of parametric cell-to-cell variation of 15 different cell parameters and the correlations between parameters based on 1100 examined cells, and found it necessary to characterize variation in ohmic impedance when determining cell-to-cell variation. Dubarry *et al*.^27,28^ have conducted both statistical and electrochemical analyses to characterize cell-to-cell variations, and found that the cell capacity, resistance and rate capability can be regarded as three independent parameters for cell variations evaluation. Furthermore, as cell parameters show different evolution trends during aging, online algorithms are required to evaluate cell-to-cell variation during aging process^15^. Kum developed an overall cell selection framework with significant improvement in fabricating a module/pack with high homogeneity using second-life cells^21^. The proposed framework consisted of cell modeling, cell testing, parameter estimation technique, and a filtering algorithm. The screening result was effective, but multiple parameters were involved in this framework and the detection algorithm is complicated, making it challenging for online operation. Several approaches have also been proposed based on precise characterization of the electrochemical performance on individual cells. However, the measurement precision is a considerable technical barrier in these cell screening methods^29–37^. The major barrier for single cell approach is the difficulty in precisely measuring or estimating cell capacity, which involves the integration of measured current containing electronic noise over an extended period of charging or discharging. High precision equipment with a high-end current source is usually required for accurate cell-to-cell homogeneity evaluation^32,38^. An excellent implementation of this strategy is the development of high precision columbic efficiency (HPCE) measurement, which is only suitable for small scale deployment mainly due to its extremely high cost^39,40^. As a result, a facile, precise and less parameter involved cell-to-cell variation evaluation process would be ideal for estimation or fabrication of a reliable stationary or portable power system.

Therefore, a facile and precise evaluation method named as “Connection in Series” or “CiS”, was proposed to evaluate the cell-to-cell variation in this work. In the CiS method, cells are evaluated in series connection, and only the cell voltages are measured for cell consistency evaluation. CiS was demonstrated to be effective for cell-to-cell variation analysis for commercial grade batteries. The simplicity, reliability and availability of CiS process for cell selection with high homogeneity was verified for vehicular application and for evaluation of cell homogeneity from different cell makers. The CiS-based methodology research as shown in this work can provide important knowledge for the development of algorithms for battery management systems (BMS) to evaluate the homogeneity of battery packs and to forecast potential failures on the fly.

## Results and Discussion

### The index of cell-to-cell variation

The cell-to-cell performance variation in a battery pack is traditionally indexed by the capacity, mass, direct current resistance, impedance, etc.^11^. The most important concern is that cell-to-cell performance variation exists after assembling the cells in series or parallel into a module. This has triggered a major research effort for developing a low cost, effective, reliable method to evaluate the performance homogeneity of cells in a battery pack^24^. As the cell-to-cell variation in capacity, impedance, and other parameters will be intrinsically reflected by their SOC variations in CiS approach, the key focus of CiS approach has evolved into selecting the most representative SOC values for effective and reliable evaluation.

**Figure 1:**
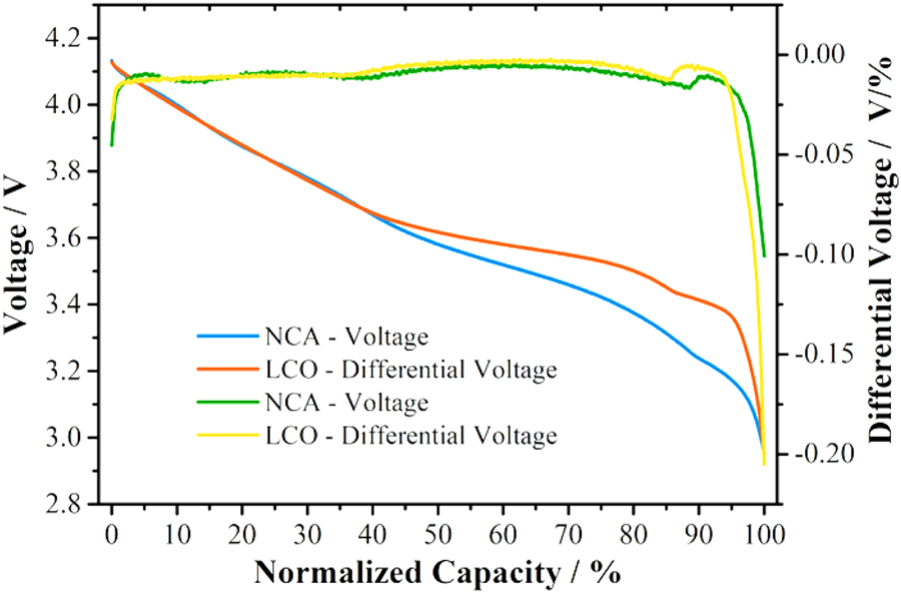
Typical discharge curves of graphite/LiNi_0.8_Co_0.15_Al_0.05_O_2_(NCA) and graphite/LiCoO_2_(LCO) cells.

As is well known, the slopes of discharge curves are the most sensitive at the end of charging/discharging. For example, Fig. 1 shows the typical discharge curves of graphite/LiNi_0.8_Co_0.15_Al_0.05_O_2_(NCA) and graphite/LiCoO_2_(LCO) cells. It can be seen that a difference of 1% in practical capacity led to a significant difference in voltage at the end of discharge by 100 mV and 205 mV for NCA and LCO cells, respectively. A difference of 205 mV in voltage can be easily translated into 4.1% of practical range for typical battery measurement equipment rated as 5 V maximum range. Moreover, the variation in the instant voltage at the end of discharge is affected by both the capacity variation and resistance variation. As a result, the instant voltage at the end of discharge will be more sensitive for the detection of cell-to-cell variation than capacity and resistance for indexing the cell-to-cell variation. Furthermore, it is known that cell capacity is calculated by multiplying the discharge duration by the coulombic current, and strongly depends on the precision of the current passing through the cell. To eliminate the discrepancy resulting from the coulombic current measurement, a Connection-in-Series (CiS) approach was proposed here to precisely screen the cell-to-cell variation. In this method, the homogeneity of cells was effectively indexed by the voltage at the end of discharge, and additionally by the voltage at the end of charge and by the voltage after a period of rest after discharge^24^. In CiS configuration, the measurement was carried out with identical amount of charge delivered to the cells in series, and the voltage data were acquired almost at the same time. The screening index of cell-to-cell variation transformed from current capacities to voltages, achieving more precise and less expensive screening of cell homogeneity. Besides the significant advantage for high precision with low cost, the methodology research of CiS-based configuration can also provide seamless knowledge integration into the development of smart battery management system (BMS). This is because the CiS configuration has already been implemented into large battery packs, and the high precision voltage tracking for each cell is the base for the BMS.

### Comparison of CiS and state-of-art test

**Figure 2:**
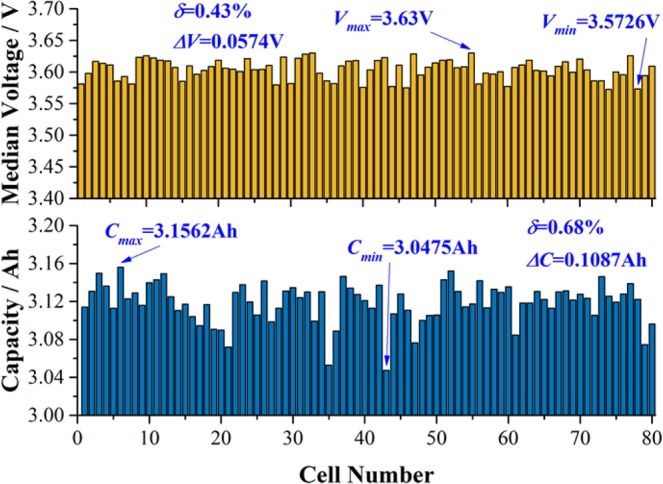
The capacities (the black dots and line) and median voltages (the blue dots and line) of 80 Panasonic cells measured during discharging at 0.2 C. The relative standard deviation (*δ*) for capacities (*Q*) was calculated to be 0.63%, and that for median voltage (*V*_*mid*_) was 0.43%.

For this test, 80 cells (NCR18650BE) purchased from Panasonic, recognized as the top battery maker with high quality, were evaluated to compare the difference between CiS and state-of-the-art. For state-of-the-art test, both capacity (*Q*) and median voltage (*V*_*mid*_, the voltages at the middle of capacity) of each cell were recorded as indexes for cell-to-cell variation. The results are shown in Fig. 2. The maximum and minimum values for median voltages (*V*_*max*_ and *V*_*min*_) were 3.63 V and 3.57 V, respectively, with a difference (*∆V*_*mid*_, *∆V*_*mid*_ = *V*_*max*_ − *V*_*min*_) of 0.057 V. The maximum and minimum capacities (*Q*_*max*_ and *Q*_*min*_) were 3.156 mAh and 3.053 mAh, respectively, which differed from each other by only 0.103 mAh (*∆Q*, *∆Q* = *Q*_*max*_ − *Q*_*min*_). The relative standard deviation (*δ*) for *Q* was calculated to be 0.63%, and that for *V*_*mid*_ was 0.43%. They were all lower than 1%, indicating excellent cell homogeneity.

**Figure 3:**
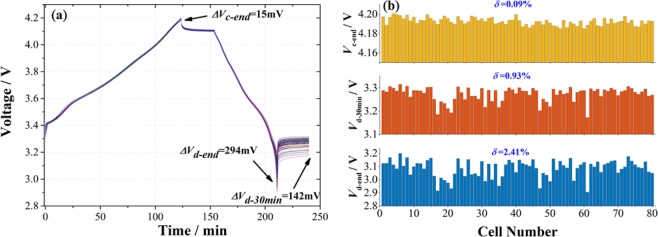
The parameter analysis of 80 cells from Panasonic by CiS. (**a**) discharge/charge curves; (**b**) The statistics of the voltages at the end of charge (*V*_*c-end*_), at the end of discharge (*V*_*d-end*_) and at the point of 30 min rest after discharge (*V*_*d-30min*_), respectively. Their relative standard deviations (*δ*) were 0.09%, 0.93% and 2.14%, respectively. The instrument error was 0.5% for 5 V range measurement.

For CiS test, these 80 cells were connected in series for charge/discharge, and experienced the same current, same duration and same capacity. The cell voltage was the only measured parameter, as shown in Fig. 3. Cell voltages at three states were investigated, i.e. the instant voltage at the end of charge (*V*_*c-end*_), the instant voltage at the end of discharge (*V*_*d-end*_), and the voltage after the end of discharge for 30 min (*V*_*d-30min*_). The results are shown in Fig. 3. *∆V*_*c-end*_, *∆V*_*d-end*_ and *∆V*_*d-30min*_ were 0.015 V, 0.294 V and 0.142 V, respectively. The relative standard deviations (*δ*) of *V*_*c-end*_, *V*_*d-end*_ and *V*_*d-30min*_ were 0.09%, 2.14% and 0.93%, respectively. The SOCs of all cells were the same (0%) at the very beginning, and all the cells were charged the same capacities in series, reaching around 100%SOC. Therefore, a very small relative standard deviation (*δ*=0.09%) of *V*_*c-end*_ was observed. The difference in *V*_*c-end*_ was mainly caused by the precision of the instrument, uneven self-discharge and impedance variation. The 15 mV difference indicates only 0.3% relative error, which is within the instrument error for 5 V range measurement (0.5%). *δ* for *V*_*d-30min*_ was 0.93%, right between *δ* = 0.43% for *V*_*mid*_ and *δ* = 2.14% for *V*_*d-end*_. This result will be applied in the next section.

**Figure 4:**
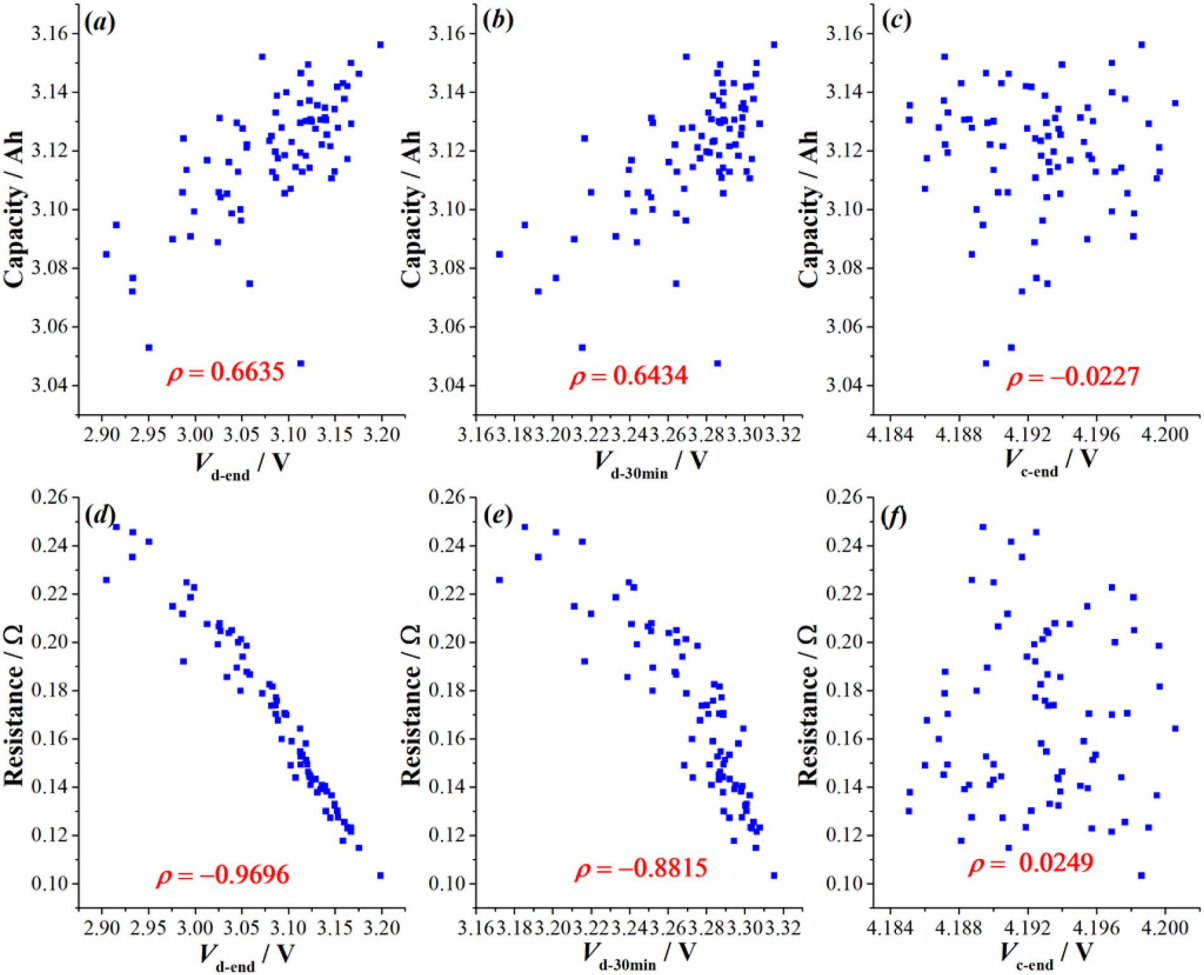
Correlations between cell capacity and the three voltage indexes measured by CiS method. (**a**–**c**) Correlations between cell capacity and *V*_*d-end*_, *V*_*d-30min*_ and *V*_*c-end*_; (**d**–**f**) Correlations between cell resistance and *V*_*d-end*_, *V*_*d-30min*_ and *V*_*c-end*_; Cell resistance was calculated by dividing the difference between *V*_*d-end*_ and *V*_*d-60s*_ by the discharge current. *V*_*d-60s*_ was the cell voltage after the end of discharge for 60 s.

As shown in Fig. 4(a–c), *V*_*d-end*_ and *V*_*d-30min*_ exhibited good correlations with cell capacity, with the correlation coefficients reaching 0.6635 and 0.6434, respectively. However, *V*_*c-end*_ showed weak correlation with cell capacity. Moreover, correlations between cell resistance and *V*_*d-end*_, *V*_*d-30min*_ and *V*_*c-end*_ were also presented in Fig. 4(d–f), where the resistance was calculated by dividing the difference between *V*_*d-end*_ and *V*_*d-60s*_ by the discharge current. *V*_*d-60s*_ was the cell voltage after the end of discharge for 60 s. *V*_*d-end*_ and *V*_*d-30min*_ also showed strong correlations with cell resistance. Therefore, we can conclude that *V*_*d-end*_ and *V*_*d-30min*_ measured by the proposed CiS method do have good correlations with cell capacity and resistance, and thus can be applied to evaluate cell-to-cell variations. Moreover, we can find that *∆V*_*d-end*_ and its *δ* were very large, being 294 mV and 2.14% respectively. Compared with state-of-the-art test results (*δ* = 0.63% for *Q* and *δ* = 0.43% for *V*_*mid*_), *V*_*d-end*_ was more sensitive to the cell-to-cell variation for the same group of cells. This verified the effectiveness of CiS and validity of *V*_*d-end*_ as an ideal index for cell-to-cell variation.

### CiS application I. Time dependence of cell-to-cell variation

**Figure 5:**
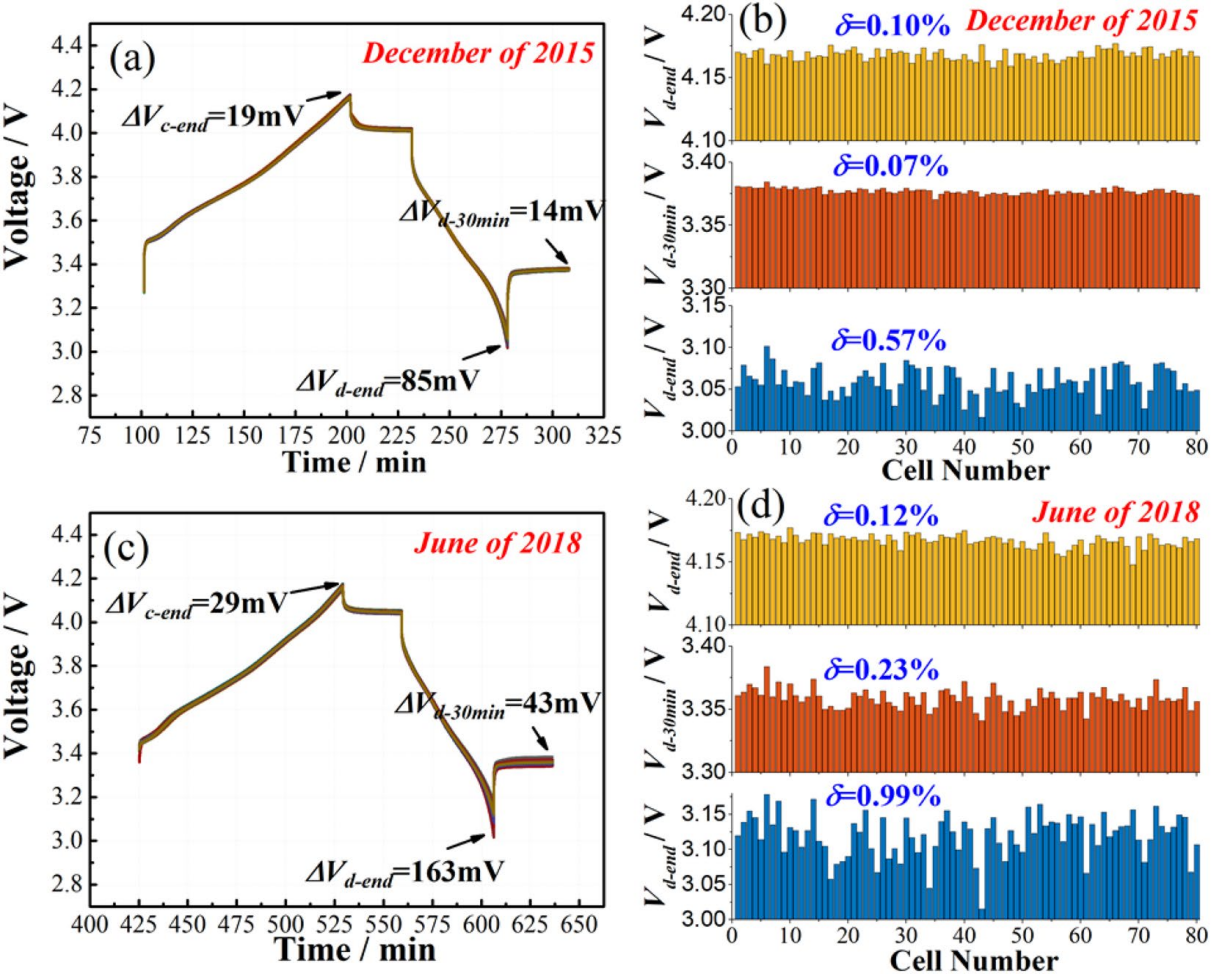
The homogeneity screening parameters of 80 cells after rest for 2.5 years. (**a,c**) charge(0.5 C)/discharge(1 C) curves of 80 cells which were connected in series; (**b,d**) The statistics of *V*_*c-end*_, *V*_*d-30min*_ and *V*_*d-end*_, respectively; (**a,b**) the parameters collected in December of 2015; (**c,d**) the parameters collected in June of 2018. When discharge/charge was at 2 C, the cells showed relative standard deviations (*δ*) of 0.09%, 0.93% and 2.14% for *V*_*c-end*_, *V*_*d-30min*_ and *V*_*d-end*_, respectively.

Due to the high sensitivity of homogeneity evaluation by CiS, the proposed CiS method was used to evaluate the possible aging differences between high quality cells shelved for 2.5 years. For this test, 80 cells from Panasonic were initially evaluated in December of 2015 (as shown in Figs. 2 and 3), and evaluated again in June of 2018. During the period between two evaluations, the cells were stored in individual packages at fully charged state at 25 °C, with the environment humidity controlled below 30%. The results are presented in Fig. 5. The same cells were tested under the same conditions, and the only change was that the two tests were 2.5 years apart. Interestingly, all the indexes for homogeneity increased. The *δ* value for *V*_*c-end*_ increased slightly from 0.10% to 0.12%. *δ* for *V*_*d-30min*_ increased considerably from 0.07% to 0.23%, but was still very small. *δ* for *V*_*d-end*_ nearly doubled from 0.57% to 0.99%. Moreover, *∆V*_*d-end*_ also increased from 85 mV to 163 mV, equivalent to the capacity difference increase from 0.13% to 0.26% when calculated by the principle shown in Fig. 1. Therefore, the CiS method can also applied to track the evolution of cell variations, and provides the basic information for cell equalization algorithm^41^.

Considering the correlation between aging speed and internal chemistry, it is reasonable that cells with similar chemical/physical states at certain time point will experience different aging speeds under the same environmental stress (such as electric stress, temperature stress, mechanical stress) and present varied chemical/physical states in the following time points^18,42,43^. In this study, cell degradation under room temperature storage was mainly caused by the SEI film formation side reaction, which is influenced by the surface area of the anode active material, the thickness of the anode and the original SEI film formed during the preparation process^44,45^. Therefore, although the cells with similar chemical/physical states were degraded under the same operation conditions, they will still experience different aging speeds over a long time period. In this sense, time is effective to amplify the cell-to cell variation. The test presented in this section indicated that a slight change in cell parameters can result in subtle differences that cannot be detected at fresh state. Moreover, this test can be applied for evaluation of battery quality from different battery makers. This may lead to a standard test protocol for battery quality evaluation, which is still a challenge in vehicular application.

### CiS application II. Rate dependence of cell-to-cell variation

Both thermodynamic and kinetic factors contribute to the cell-to-cell variations^2,10–12,27,46,47^, and kinetic factors are generally presented as impedances in battery. In this sense, the rate dependence of cell-to-cell variations will help to identify the influencing factors for battery kinetics introduced during manufacturing^11^. However, the cell-to-cell variation dependence on C-rate is unclear. On one hand, the subtle variation in impedance can be magnified in numerical value at high C-rate. On the other hand, high C-rate operation may cause temperature rise inside the battery, which is known to help decrease the battery impedance. That is, high C-rate may lead to impedance reduction in numerical value. Furthermore, high rate charge/discharge implies high measurement error for normal charge/discharge instrument, which affects the analysis of cell-to-cell variation. Here, the preferable C-rate for CiS was determined, and the possible application of CiS at different C-rates was also presented. CiS approach was employed to investigate the rate dependence of cell-to-cell variations. The evaluation of cells from different manufacturers was further discussed.

**Figure 6:**
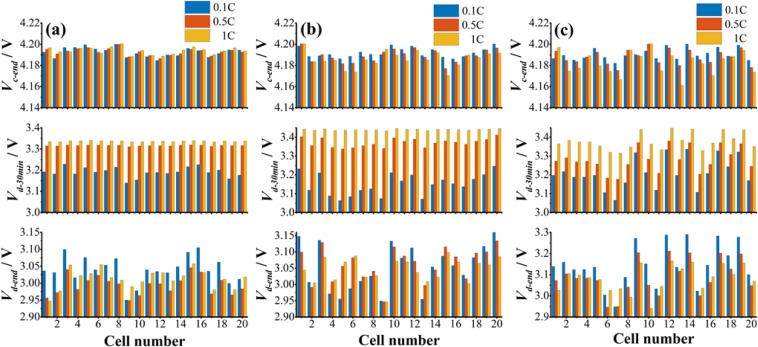
The statistics of the *V*_*c-end*_ (3 lines at the top), *V*_*d-end*_ (3 lines at the bottom) and *V*_*d-30min*_ (3 lines in the middle) of 20 cell-series modules operated at various C-rates with cells produced by different battery makers. (**a**) 20 cells from Panasonic, NCR18650BE, 3.0Ah; (**b**) 20 cells from LG Chem, 18650 B4, 2.6Ah; (**c**) 20 cells from Lishen, LR1865SK, 2.6Ah. The tests were carried out at 0.1 (black), 0.5 (red) and 1.0 (blue) C-rate, respectively. The tested 20 cells were the most uniform ones screened by CiS from stochastic 80 cells, respectively.

The cells produced by Panasonic (NCR18650BE, 3.0 Ah), LG Chem (18650 B4, 2.6 Ah) and Lishen (LR1865SK, 2.6 Ah) were evaluated respectively. Firstly, 20 cells with good consistency were selected among 80 cells by CiS at 0.1 C-rate according to *δ*. Then, cell-to-cell variation was further checked at 0.5 and 1 C-rate. The results are shown in Fig. 6. All the tests confirmed that *V*_*d-end*_ was the most sensitive parameter for cell-to-cell variation. The charge tests were stopped when any cell reached 4.2 V. It was found that each *V*_*c-end*_ was very close to 4.2 V, indicating that the uniformity was good for charging process. The 20 cells of any brand selected according to *V*_*d-end*_ can ensure high homogeneity of *V*_*c-end*_, proving the effectiveness of CiS. It is known that high homogeneity of *V*_*c-end*_ is important to avoid overcharging in a module or pack configuration.

For *V*_*d-30min*_, cell homogeneity at different C-rates can be roughly determined by the fluctuation range of the statistical curves. Then, it was found that cells from Panasonic exhibited good homogeneity at both 0.5 and 1.0 C-rate, as shown in the middle of Fig. 6(a). Cells from LG Chem showed good consistency only at 1.0 C-rate, as shown in Fig. 6(b). However, the homogeneity of cells from Lishen was worse compared to the other two. In this sense, the ranking order of homogeneity was Panasonic, LG Chem and Lishen.

Interestingly, the sequence of cell number changed in homogeneity screening by different C-rates, according to voltage value. For example, in Fig. 6(a), the *V*_*d-end*_ of cell No.6 was higher than that of the two adjacent cells at 1 C rate, but it was lower at 0.1 C rate. That might be induced by variations in cell rate capability. Cell No.6 had a smaller capacity than the two adjacent cells and thus showed a lower *V*_*d-end*_ at 0.1 C. Inversely, cell No.6 exhibited a larger capacity when C-rate increased to 1 C, indicating that cell No.6 might has better rate capability than the adjacent cells^27,28^. The cells from LG Chem and Lishen also showed similar behavior, as presented in Fig. 6(b,c). This indicates that the rate dependence of cell-to-cell variation may provide more information about the battery performance, such as variations in rate capability, which is an important index for cell-to-cell variations^27,28^.

**Figure 7:**
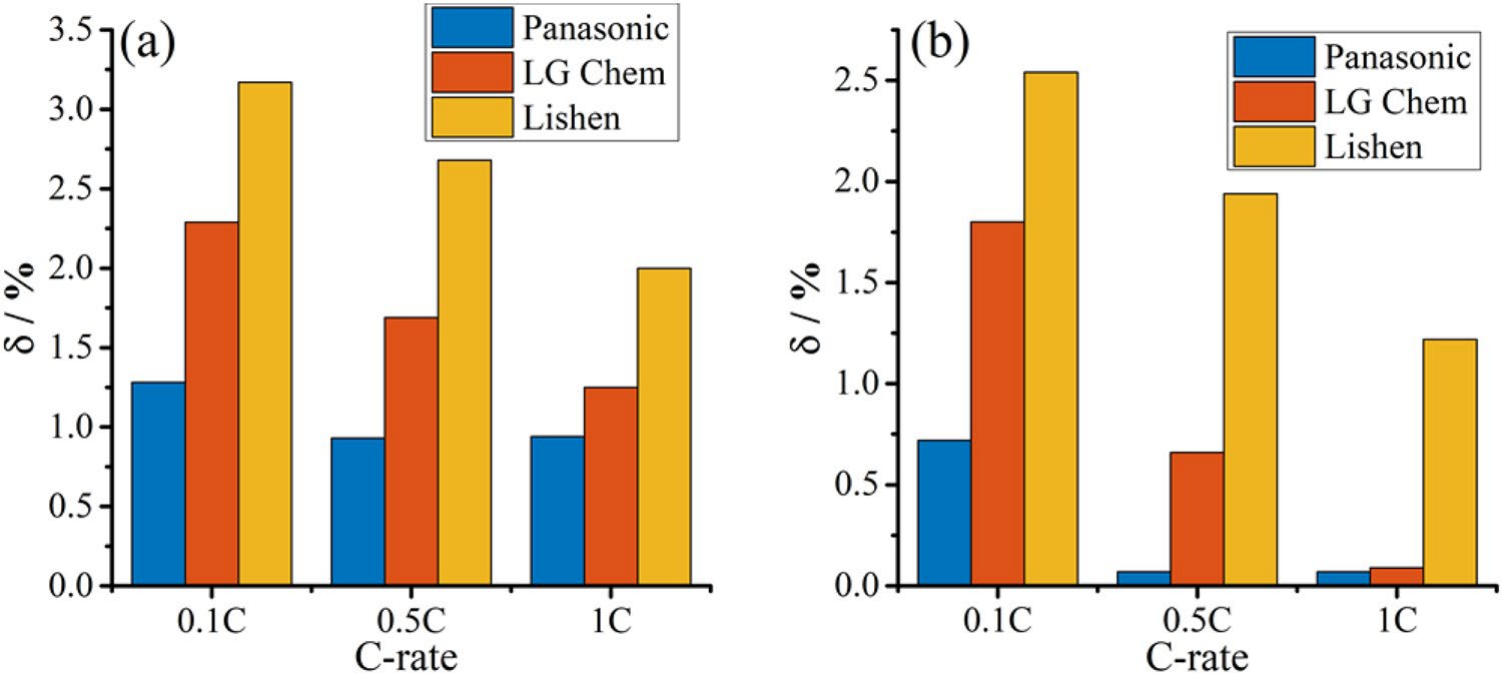
The relative standard deviations (*δ*) of cell parameters at different C-rates by CiS. (**a**) *V*_*d-end*_; (**b**) *V*_*d-30min*_. For each battery maker, 20 cells were screened from 80 cells by CiS, respectively.

Quantitatively, relative standard deviation (*δ*) is proposed as an appropriate index for homogeneity. In statistics, *δ* is also known as the coefficient of variation, a standardized measure of dispersion for a probability distribution^48,49^. It was observed that the evolution trend of *δ* for *V*_*d-end*_ was similar to that for *V*_*d-30min*_, as shown in Fig. 7(a,b). However, the former was larger (more sensitive) than the latter. The relative standard deviation also confirmed once again that *V*_*d-end*_ is a suitable index for cell-to-cell variation.

The cells from Panasonic exhibited the lowest *δ* values at all C-rates, and the *δ* values of *V*_*d-end*_ and *V*_*d-30min*_ were both less than 1.5%. The cells from LG Chem showed larger variations with the *δ* of *V*_*d-end*_ and *V*_*d-30min*_ increasing to more than 2.0% at 0.1 C. The cells from Lishen showed the worst homogeneity with largest *δ* at all C-rates. Figure 7 indicates quantitatively that the ranking order of cell homogeneity was Panasonic, LG Chem and Lishen. It is well known that the cells from Panasonic have top quality, featuring homogeneous slurry, uniform coating of electrodes, etc. This is consistent with the above results.

**Figure 8:**
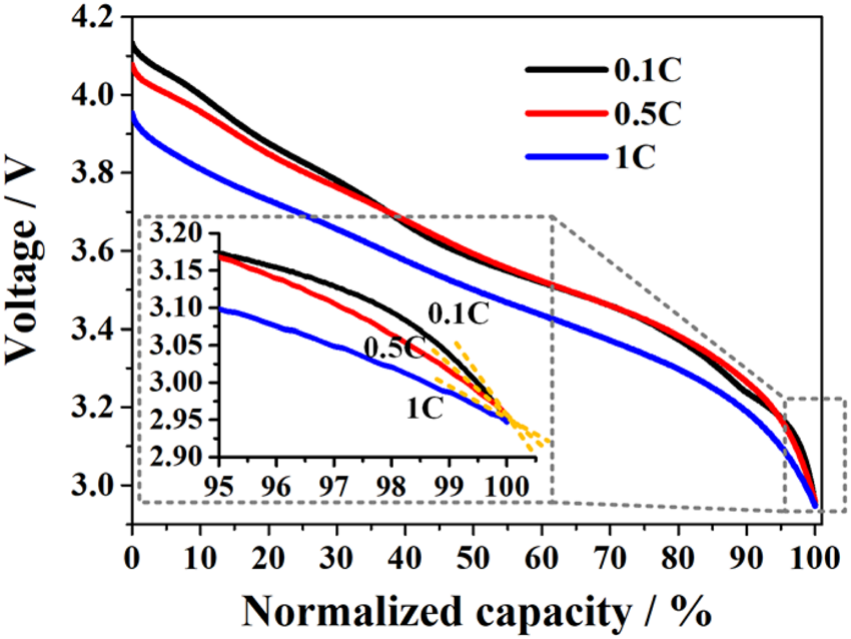
Discharge voltage curves of Panasonic cells at different C-rates.

Furthermore, the cells from the three manufacturers all exhibited increasing *δ* values with decrease in C-rate. The changes in *δ* with C-rates can be explained by the different slopes of the voltage curves at different C-rates, as presented in Fig. 8. The discharge voltage curves exhibited small slope with the increase in C-rate due to the influence of polarization. Thus, *V*_*d-end*_ became less sensitive to cell-to-cell variation as C-rate increased. Moreover, the changes of *δ* with C-rates can help to evaluate the rate capabilities of the cells from different manufacturers. According to Fig. 8, a larger decrease in the *δ* of *V*_*d-end*_ with the increase of C-rates indicated a larger increase of polarization and thus worse rate capability. Form the results in Fig. 7(a), we can observe that the Panasonic cells have the best rate capabilities among the three manufacturers. A index named *RC* was also defined as the ratio between the capacity at 1 C and 0.1 C to evaluate rate capability referring to ref. ^17^. The Panasonic cell exhibited the best rate capability with a *RC* ratio of 83.7%, while the *RC* ratios of LG Chem and Lishen cells were 79.6% and 80.4%, respectively, consistent with results from the CiS method. This finding also enriches the understanding of rate dependence of cell-to-cell variations^11^.

## Conclusions

Cell homogeneity is critical for performance and durability of energy storage systems. The state-of-the-art evaluation of cell homogeneity based on individual cell performance involves intertwined parameters (such as capacity and impedance) and a large number of measurements. Based on the charge/discharge characteristics of lithium-ion batteries, a CiS method that indexes cell-to-cell variations only by a state voltage was proposed in this paper. In detail, the voltage at the end of discharge *V*_*d-end*_ was demonstrated to be a sensitive index for cell-to-cell variation, and the relative standard deviation (*δ*) was proposed to be an appropriate index for cell homogeneity in parameter processing. Low C-rate was preferred for CiS, as the cell homogeneity decreased with decrease in C-rate. Compared with the state-of-the-art evaluation method^11^, the proposed CiS approach can save equipment cost, manpower, analysis test and data processing. Investigations on the variation of cell homogeneity with storage duration showed that cell homogeneity became worse during aging even though the cells started from quite similar physical and chemical states. Besides, the dependence of cell homogeneity on C-rate can be used as an index for quality control or quality evaluation. The information may help battery makers to improve cell design, manufacturing and quality control. CiS was shown to be a facile and highly precise approach for evaluating and tracking the consistency of lithium-ion batteries. It can not only be developed as a universal standard test protocol of cell-to-cell variation for vehicular and stationary energy storage application, but also have possible applications in cell screening.

## Materials and Methods

### Materials

Cylinder cells (18650 type) were purchased from market and were not balanced before. The cell manufacturers were Panasonic (NCR18650BE, 3.0 Ah), LG Chem (18650 B4, 2.6 Ah) and Lishen (LR1865SK, 2.6 Ah), respectively. The cathode material was LiNi_0.8_Co_0.15_Al_0.05_O_2_(NCA) for Panasonic cell, and was LiCoO_2_ (LCO) for LG Chem and Lishen cells. The anode material of the three kinds of cells was artificial graphite. The separators were all polypropylene. Neware test system was used to perform charge-discharge tests, and Arbin EVTS battery testing system was used to record cell voltage during charge-discharge.

### State-of-the-art test method

The capacities and median voltages of Panasonic cells were measured individually before the CiS tests at 25 °C according to the product specification. The charge protocol was CC-CV with 1/5 C reaching an upper voltage limit of 4.2 V, and the cutting-off current was 0.05 C. After a rest period of 30 min, the cells were discharged with a constant current 1/3 C to 2.5 V.

### CiS test method

**Figure 9:**
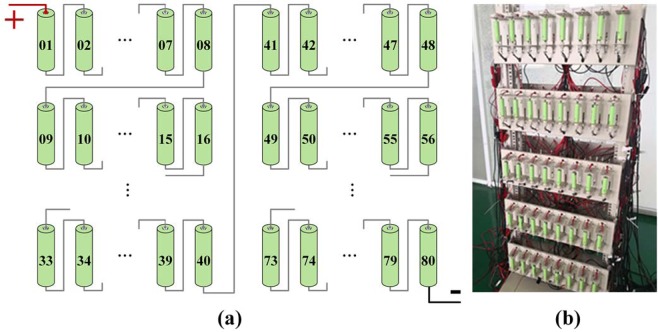
The CiS test method. (**a**) schematic of the CiS test method; (**b**) photo of the cells connected and tested in series.

All cells with 100%SOC were connected in series. The voltage of every single cell was recorded by Arbin EVTS battery testing system (accuracy of voltage control was ±0.1% FSR). The homogeneity of 80 Panasonic cells at 2.5 year intervals was investigated by CiS. As shown in Fig. 9, 80 cells were connected and tested in series. During the tests, the same amount of charge/discharge capacity passed through the 80 cells, and the voltage of each cell was measured simultaneously. Cells were firstly discharged at 0.2C-rate until any single voltage reached 2.95 V. Then, the cells were rested 10 min and were charged at 0.5C-rate when any single voltage reached 4.2 V. The discharging process consisted of discharging at 1C-rate until any single voltage reached 3.0 V, and then a 30 min rest. The C-rate was determined by nominal capacity of the tested cell. *X* C represents the current that can fully discharge the cell from 100%SOC to 0%SOC in 1/*X* hour. 1 C was 3.0 A for Panasonic NCR18650BE cells (3.0Ah), and 2.6 A for both LG Chem 18650B4 cells (2.6Ah) and Lishen LR1865SK cells (2.6Ah). Three instant voltages, i.e. the end of charging (*V*_*c-end*_), discharging (*V*_*d-end*_) and discharging for 30 min (*V*_*d-30min*_), in the process were recorded and their discrete factors were calculated as the criteria for homogeneity evaluation.

To investigate the dependence of consistency to rate, 20 cells with the best homogeneity were selected among 80 cells for the three kinds of cells respectively. In this process, the charge/discharge rate was 0.1 C. Then the consistency of each kind of 20 cells was measured using CiS method at 0.5 and 1 C-rate for both charge and discharge at 25 °C. The test protocol was as follows:

1. all cells were charged to 100% SOC. (the charge protocol refers to the battery specifications provided by manufacturer: charging to 4.2 V with 0.3 C constant current (CC) and then constant voltage (CV) charging with cut-off current of 0.05 C);
2. the 20 cells were connected in series to form a module, and the voltages of individual cells were recorded;
3. the module was discharged at 0.1 C to 3.0 V of any single cell;
4. rest for 30 min;
5. the module was then charged at 0.1 C up to 4.2 V of any single cell; then steps 3, 4, and 5 were successively repeated at 0.5 C and 1.0 C, respectively.
